# Proboscidean Mitogenomics: Chronology and Mode of Elephant Evolution Using Mastodon as Outgroup

**DOI:** 10.1371/journal.pbio.0050207

**Published:** 2007-07-24

**Authors:** Nadin Rohland, Anna-Sapfo Malaspinas, Joshua L Pollack, Montgomery Slatkin, Paul Matheus, Michael Hofreiter

**Affiliations:** 1 Max Planck Institute for Evolutionary Anthropology, Leipzig, Germany; 2 Department of Integrative Biology, University of California Berkeley, Berkeley, California, United States of America; 3 Department of Zoology and Animal Biology, University of Geneva, Geneva, Switzerland; 4 Alaska Quaternary Center, University of Alaska Fairbanks, Fairbanks, Alaska, United States of America; Massey University, New Zealand

## Abstract

We have sequenced the complete mitochondrial genome of the extinct American mastodon *(Mammut americanum)* from an Alaskan fossil that is between 50,000 and 130,000 y old, extending the age range of genomic analyses by almost a complete glacial cycle. The sequence we obtained is substantially different from previously reported partial mastodon mitochondrial DNA sequences. By comparing those partial sequences to other proboscidean sequences, we conclude that we have obtained the first sequence of mastodon DNA ever reported. Using the sequence of the mastodon, which diverged 24–28 million years ago (mya) from the Elephantidae lineage, as an outgroup, we infer that the ancestors of African elephants diverged from the lineage leading to mammoths and Asian elephants approximately 7.6 mya and that mammoths and Asian elephants diverged approximately 6.7 mya. We also conclude that the nuclear genomes of the African savannah and forest elephants diverged approximately 4.0 mya, supporting the view that these two groups represent different species. Finally, we found the mitochondrial mutation rate of proboscideans to be roughly half of the rate in primates during at least the last 24 million years.

## Author Summary

We determined the complete mitochondrial genome of the mastodon *(Mammut americanum),* a recently extinct relative of the living elephants that diverged about 26 million years ago. We obtained the sequence from a tooth dated to 50,000–130,000 years ago, increasing the specimen age for which such palaeogenomic analyses have been done by almost a complete glacial cycle. Using this sequence, together with mitochondrial genome sequences from two African elephants, two Asian elephants, and two woolly mammoths (all of which have been previously sequenced), we show that mammoths are more closely related to Asian than to African elephants. Moreover, we used a calibration point lying outside the Elephantidae radiation (elephants and mammoths), which enabled us to estimate accurately the time of divergence of African elephants from Asian elephants and mammoths (about 7.6 million years ago) and the time of divergence between mammoths and Asian elephants (about 6.7 million years ago). These dates are strikingly similar to the divergence time for humans, chimpanzees, and gorillas, and raise the possibility that the speciation of mammoth and elephants and of humans and African great apes had a common cause. Despite the similarity in divergence times, the substitution rate within primates is more than twice as high as in proboscideans.

## Abstract 3

The first complete mitochondrial DNA genome for the mastodon extends the age range for genomic analyses by almost a complete glacial cycle and resolves the relationships among African and Asian elephants and mammoths.

## Introduction

An accurate and well-supported phylogeny is the basis for understanding the evolution of species. With the appropriate and adequate amount of data, it is possible not only to determine relationships among species, but also to date divergence events between lineages. In turn, divergence events can be correlated to environmental changes recorded in the fossil record to help understand mechanisms driving evolution. The power of these correlations, and arguments for particular environmental mechanisms driving evolutionary change, increases when the pattern is repeated across multiple taxa.

Sequencing complete mitochondrial genomes has become a powerful tool in the investigation of phylogenetic relationships among animal groups, principally mammals, birds, and fishes. Despite this potential, and the proliferation of ancient mitochondrial DNA (mtDNA) research, geneticists so far have succeeded in sequencing complete mitochondrial genomes from ancient DNA for only a few extinct species of moas [1,2] and the woolly mammoth [3,4]. For both groups, the complete sequences resolved long-standing evolutionary questions, which argues for an extension of such analyses to other species.

The living elephants comprise the last survivors of the Elephantidae, a once-flourishing sub-group of the Order Proboscidea that lived throughout much of Africa, Eurasia, and the Americas [5]. The evolution of Elephantidae, which includes the recently extinct mammoths, has been extensively studied in recent years using both modern and ancient sequences of mtDNA and nuclear DNA (nuDNA). For example, nuDNA sequences have been used to argue that the African forest elephant is a valid species *(Loxodonta cyclotis),* distinct from the African savannah elephant *(L. africana)* [6], with the two species having diverged by 2.6 million years ago (mya), though this view is disputed [7].

Two recent studies reported complete mtDNA genomes from the woolly mammoth *(Mammuthus primigenius)* [3,4] that provided strong evidence that mammoths were more closely related to Asian elephants than to African elephants. However, another study analyzing several nuDNA segments cautioned that the lack of a closely related outgroup is a problem in phylogenetic analyses of Elephantidae and argued that the relationship between mammoth and the living elephants is still unresolved [8]. To date, all genetically based analyses of elephant phylogenies have used dugong *(Dugong dugon)* and hyrax *(Procavia capensis)* as outgroups. These are the nearest living relatives of elephants, but they diverged from proboscideans some 65 mya [5,9], which severely limits their power as effective outgroups for assessing elephant genetic data.

In contrast, ancestors of the American mastodon, *Mammut americanum,* diverged from the Elephantidae lineage no earlier than 28.3 mya [10], which would make mastodon a much more appropriate outgroup for the Elephantidae. Moreover, mastodon fossils preserved in permafrost and dating to the late Pleistocene have been recovered in eastern Beringia (Alaska and Yukon). Their young age and preservation in permafrost means Beringian mastodon are excellent candidates for ancient DNA analyses [11,12]. The good biochemical preservation of mastodon samples was appreciated early in the study of ancient DNA. In 1985, Shoshani and colleagues determined the immunological distances of albumins among mammoth, mastodon, and African and Asian elephant [13].

We report here what is to our knowledge the first complete mitochondrial genome for mastodon. The sequence was derived from a tooth collected in northern Alaska, where Pleistocene bones are well-preserved in permafrost. Collagen from the root of the tooth was radiocarbon-dated to more than 50,000 y before present (BP), but the geological provenance suggests it is not older than 130,000 y (see Materials and Methods). This extremely old and complete sequence is of interest in its own right, but it also can be used to help resolve existing debates concerning the phylogeny of the Elephantidae and allows us to evaluate rates of molecular evolution within the Proboscidea, because it finally provides an appropriate outgroup for such analyses. Here, we use the mastodon mtDNA sequence to resolve relationships among African elephants, Asian elephants, and mammoths and to more accurately date their divergence times.

## Results/Discussion

### Sequence Determination and Characteristics

**Figure 1:**
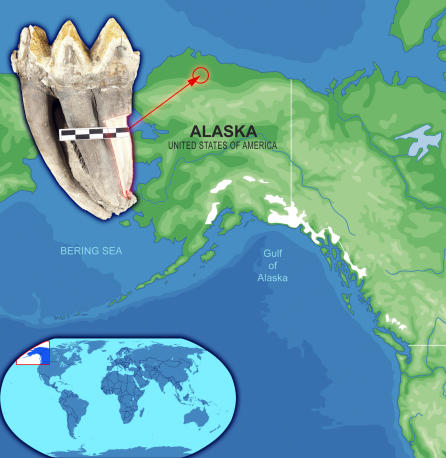
Appearance and Location of Origin of the Mastodon Tooth (IK-99–237) Used for Sequencing of the mtDNA Genome The scale bar shows centimeters.

To determine the mastodon mtDNA genome sequence, we used 78 primer pairs, separated into two sets of 39 pairs each, on DNA extract from a mastodon molar originating from northern Alaska (Figure 1) and performed quadruplicate amplifications for each primer pair. In the first round of amplification, 48 primer pairs yielded at least one positive result, and for 36 primer pairs all four attempts were positive. To obtain the remaining fragments, we used four consecutive rounds of redesigning primers (see Protocol S1 for details). In each round, whenever possible, we used the mastodon sequences from flanking fragments as the basis for primer design. With three primer pairs, PCR controls or extraction controls yielded products of the correct size. All three cases of contamination occurred with primers for which it was not possible to design the primers in a way that they selected against the amplification of human DNA. The resulting products were cloned and sequenced and turned out to be exclusively of human origin. Two out of six clones obtained from a single amplification from mastodon extract using one of these primer pairs were also of human origin. We did not replicate part of the mastodon sequence in an independent laboratory, following the argument we have made previously [14], and in contrast to Cooper and Poinar [15], who argued that this is necessary in all ancient DNA studies. None of the determined sequence fragments was identical to any known sequence either from GenBank or determined previously in our laboratory. Moreover, to ensure authenticity of each individual fragment, apart from extensive internal replication, we compared each PCR fragment individually to all published sequences from GenBank and noted the similarity to both Elephantidae and hyrax and dugong mtDNA sequences. All amplified fragments showed between 78.1% and 98.6% identity to Elephantidae sequences and between 45.9% and 92.6% identity to hyrax and dugong sequences. Moreover, for individual fragments, the average identity to Elephantidae was 6.2% to 40% higher than that to hyrax and dugong. These results are consistent with the phylogenetic position of the mastodon and difficult—if not impossible—to explain by contamination. Therefore, we conclude that our results indeed represent authentic mastodon sequences.

The length of the mastodon mitochondrial genome is 16,469 bp. Thus, it is about 300–400 bp smaller than the mitochondrial sequences of the other proboscideans published to date. This difference in length is most likely an artifact due to the failure to obtain an amplification product that covers the complete tandem repeat in the control region. The mastodon mitochondrial genome contains 13 protein coding genes, 22 *tRNA* genes, two *rRNA* genes, and the control region, as expected for a placental mammal. Stop and start codons are shared with at least one of the other proboscideans for each of the protein coding genes, except *ND3, ND4,* and *ND6.* For these three genes, the mastodon sequences start with ATT, ATG, and GTG, whereas the corresponding genes of all other proboscideans start with ATC or ATA, GTG, and ATG, respectively. Differences in the annotation were observed in the other sequences notably because of stop codons being created by polyadenylation, a phenomenon widely present in mitochondrial genomes of other vertebrates [16]. The early stop of *ND4* of the woolly mammoth sequences was not observed, and none of the proteins of the mastodon seems to be truncated. With a divergence time of at least 24 million years from any living relative, the mastodon sequence shows that the multiplex PCR approach can also be applied to taxa without sequence information from closely related species.

### Comparison with Previously Published Mastodon Sequences

Partial DNA sequences that are claimed to be derived from mastodon are available from four studies. The sequences of Yang et al. [17] (228 bp of *CYTB*) and Joger et al. [18] (294 bp of *CYTB*) show substantially less divergence from mammoth and elephants than our sequence. The numbers of substitutions between their mastodon sequences and the Elephantidae are comparable to differences within the Elephantidae (results not shown). In contrast, our sequence clearly differs from the elephantid sequences. The results of Yang et al. have been recently questioned [19,20]. Debruyne et al. [19] concluded that Yang et al.'s mammoth sequence is a probable chimera of African and Asian elephant. Our study also rejects the results of Yang et al. for mastodon because the fragment of their *CYTB* mastodon sequence clusters closely within Elephantidae (see Figure S1A), as expected by the number of substitutions. For the same reason, the partial sequence of Joger et al. also seems to be derived from an Asian/African elephant contaminant (see Figure S1B). Finally, the unpublished 16S rDNA sequences obtained by Park et al. and Goldstein et al. (GenBank accession numbers AF279699 and AY028924, respectively) show 97% and 99% identity to primates using Blastn [21], which shows that these two sequences also were very likely derived from contamination.

### Phylogenetic Results

**Figure 2:**
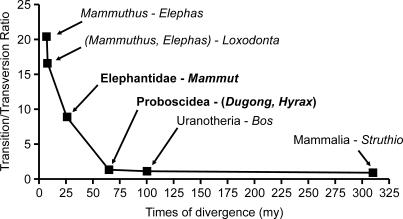
Ratio of the Number of Transitions to the Number of Transversions The six points correspond to the ratio between species of Elephantidae (first two points), between Elephantidae and *Mammut,* between Proboscidea and (*Dugong, Procavia*), between Uranotheria and *Bos,* and between Mammalia and *Struthio*. The corresponding divergence times for proboscideans are given in Table 2. Dugong and hyrax diverged from Proboscidea 65 mya [5,9]. We used 105 mya for the Uranotheria–ruminant split ([9] and citations therein). The mammalian–bird divergence was set to 313 mya (the mean value of the confidence interval of [62]; but see also [63,65]). The arrows indicate the points of interest. The uncertainty of the divergence times is not taken into account because the goal was to show the general trend.

In order to further investigate proboscidean evolution, we used the six existing whole mitochondrial genomes of Elephantidae (two African elephants, *L. africana;* two Asian elephants, *Elephas maximus;* and two woolly mammoths, Mammuthus primigenius) together with our newly obtained Mammut americanum sequence. As the mastodon lineage is evolutionarily much closer to Elephantidae than to extant outgroup species such as dugong or hyrax, multiple substitutions should not represent such a pronounced problem as is the case with the latter species. A comparison of the seven proboscidean mitochondrial genomes to those from hyrax and dugong shows that the number of substitutions separating mastodon from the Elephantidae is less than half that separating either hyrax or dugong from the Elephantidae (Table S1). Consistent with this result, the rates of synonymous (*d*_s_) and nonsynonymous (*d*_n_) substitutions are much lower when using mastodon as the outgroup (*d*_s_ = 0.4; *d*_n_ = 0.05). Using dugong or hyrax as the outgroup, *d*_s_ is 1.2, showing substitution saturation [4], and *d*_n_ is still 0.15. The transition/transversion ratio can also be used as a measure of substitution saturation [3,22]. Including the whole mitochondrial genome of the cow *(Bos taurus)* and the ostrich *(Struthio camelus)* reveals that dugong and hyrax have reached a plateau for both number of substitutions and transition/transversion ratio when compared to Elephantidae, whereas the mastodon has not (Figure 2). Thus, as previously noted [23], the mastodon represents a much better outgroup for inferring Elephantidae evolution than either dugong or hyrax.

The greater suitability of mastodon as an outgroup to the Elephantidae is further demonstrated by the results of the phylogenetic analyses. Using the mastodon sequence as an outgroup, we obtained higher support values for a sister group relationship of mammoth and Asian elephant than previous studies [3,4] and obtained the same tree topology from different methods of phylogenetic inference (Table 1). The bootstrap values for this relationship were between 94% (neighbor joining [NJ]) and 99% (maximum likelihood [ML]) with a Bayesian posterior probability of 1.00 (Table 1). These bootstrap values do not vary significantly among substitution models and are not dependent on whether the data are partitioned or unpartitioned (results not shown). Thus, at least with regard to mtDNA sequences, the relationship among mammoth and the living elephant species can no longer be seen as equivocal, as argued by some authors [8]. This result indicates that mastodon would also provide an excellent outgroup for phylogenetic analyses of Elephantidae using nuclear sequences [8] if it became possible to recover nuclear mastodon DNA sequences.

**Table 1:**
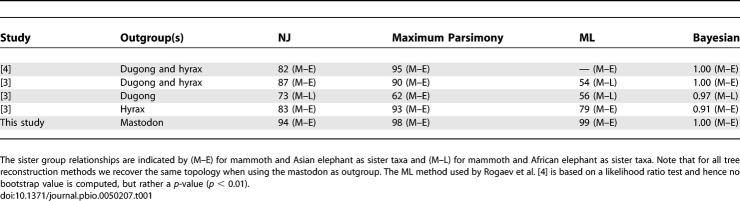
Increase of the Support (Bootstrap or Posterior Probability) When Using the Mastodon as Outgroup to Reconstruct the Elephantidae Phylogeny

**Table 2:**
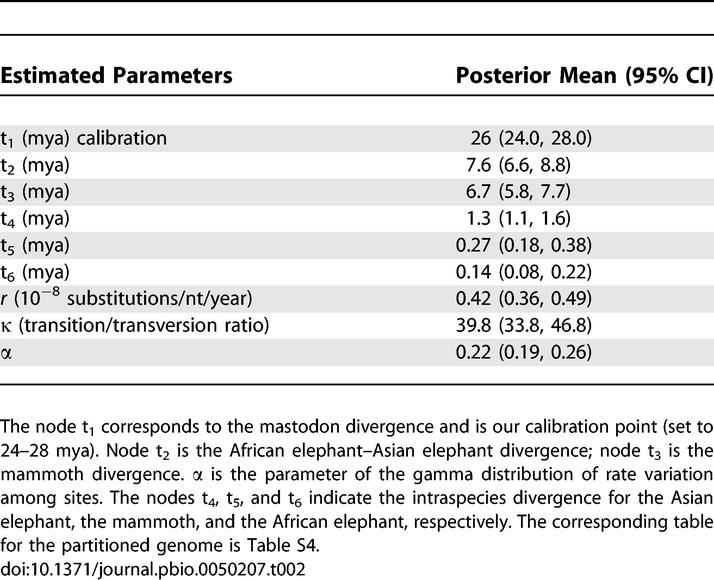
Divergence Times Calculated Using Whole Mitochondrial Genomes of the Four Proboscidean Species

**Figure 3:**
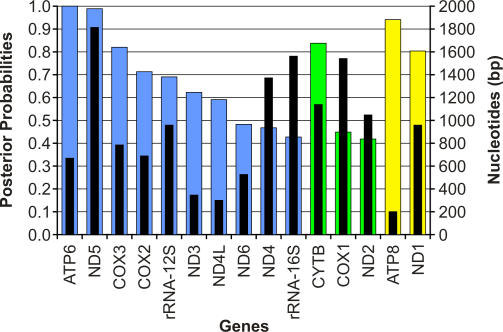
Gene-by-Gene Topology Colored bars indicate the posterior probabilities for each respective topology. Blue indicates a *Mammuthus–Elephas* sister group relationship, green indicates a *Mammuthus–Loxodonta* sister group relationship, and yellow indicates an *Elephas–Loxodonta* sister group relationship. Black bars show the length of the respective genes in base pairs.

Using our complete mtDNA mastodon sequence, we were able to employ gene-by-gene phylogenetic analyses to explain why several earlier studies found a sister group relationship between African elephants and mammoths. The reconstructed phylogeny of the Elephantidae varied widely when we used each of the 13 protein coding genes and the two *rRNAs* individually. We recovered the mammoth–Asian elephant topology for the majority of the genes, but with lower support values (44%–90% for bootstraps and 0.42–1.00 for posterior probabilities). Other genes supported different tree topologies, sometimes with high bootstrap values or Bayesian posterior probabilities (up to 90% or 1.00; see Figure 3 and Table S2). In fact, when considering NJ trees alone, the majority (eight of 15) of the single-gene analyses in fact supported an incorrect topology. Some single-gene analyses resulted in different, yet well supported topologies when hyrax and dugong were used as the outgroup instead of mastodon [4]. These results indicate that studies based on a single gene can be misleading, and long sequences may often be necessary to obtain correct phylogenies (Figures 3 and S2; Protocol S3; see also, e.g., [24–27]).

### Dating of Elephantidae Divergence Events

Unlike the sequences of the nearest living outgroups of the Elephantidae, the dugong and the hyrax, the mastodon sequence has not yet reached substitution saturation (Figure S3). Consequently, the mastodon mtDNA sequence provides better estimates of the dates of divergence within the Elephantidae. Moreover, fossil evidence constrains the divergence of hyrax and dugong from proboscideans to earlier than ∼60 mya, the date for the oldest proboscidean fossil genus, *Phosphatherium* [28]. Previous attempts to date the Elephantidae divergence either used the two very distant outgroups, with limited success because the sequences violated the molecular clock assumption [4], or used the divergence of African elephant as calibration point [3], which yields more reliable relative divergence dates within Elephantidae, but does not provide independent estimates of divergence times within this group. Thus, the mastodon sequence allows independent dating of Elephantidae divergence times based on the sequence of a well-calibrated outgroup.

**Figure 4:**
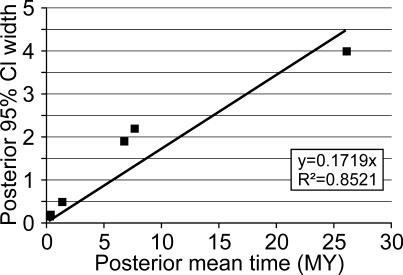
Data Saturation as Determined by the 95% CI Width as a Function of Posterior Mean Time The high correlation coefficient of this plot suggests that the sequence data are highly informative. However, it cannot be excluded that the accuracy of divergence time estimates could be further improved by additional sequence data. The graph corresponds to the whole genome analysis. The partitioned data give a slightly higher correlation coefficient of 0.89 (not shown).

**Figure 5:**
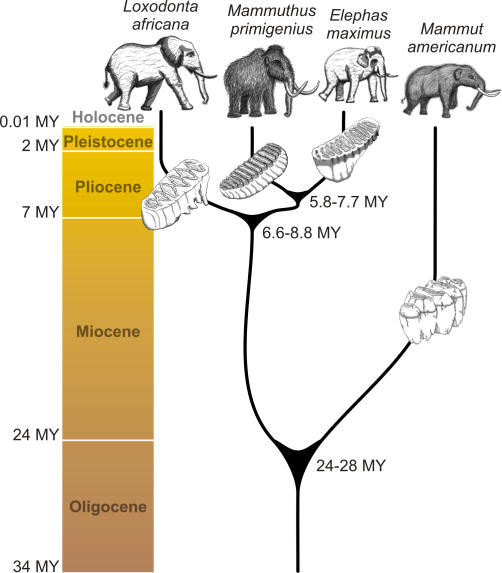
Phylogeny and Divergence Times Determined by This Study for the Four Proboscidean Species Analyzed

Using the best-fitting topology, we found the TN93 [29] model of substitution to be the simplest that fitted the data (results not shown). Using this substitution model, we were not able to reject the assumption of a molecular clock for the whole mtDNA genome sequences of the mastodon and the three Elephantidae species (2Δℓ = 6.8; *p* = 0.24). Using the Bayesian approach of Yang and Rannala [30], we evaluated the saturation level of our data by plotting the posterior means versus the width of the 95% credibility intervals (CIs) of the divergence times. The relationship is almost linear, and the coefficient of correlation of the linear regression has a value of 0.85 (Figure 4). Although a correlation of 0.85 suggests that the sequence data are highly informative it is still possible that the accuracy of divergence time estimates could be further improved by additional sequence data. When we used the paleontologically determined divergence date of 24–28 mya for mastodon [10,31], the divergence time of the African elephant turned out to be older than the 6 mya previously assumed [32]. In fact, we calculated it to have a posterior mean of 7.6 mya (95% CI 6.6 to 8.8 mya) when using the whole mitochondrial genome. The divergence between mammoth and Asian elephant also moves back in time to 6.7 mya (CI 5.8 to 7.7 mya; Figure 5; Table 2). Both dates are concordant with the presence of African elephant and Asian elephant fossils by 5.4–7.3 and 5.2–6.7 mya [33], respectively. Only about a million years separates the two divergence events, which is less than in humans, chimpanzees, and gorillas [34], for which extensive lineage sorting has been found [35,36]. Hence, we expect lineage sorting for the nuclear genome to also be problematic for Elephantidae, as claimed by Capelli et al. [8].

Our new estimates make the divergence of mammoth and African and Asian elephants even closer in time to the divergence of humans, chimpanzees, and gorillas, and other mammalian taxa. A number of environmental changes were occurring globally at that time, including the spread of grasslands and an increase in C_4_ plant biomass [37,38]. Further efforts should be given to studying the relationship between environmental changes and these phylogenetic events during the late Miocene.

Finally, our revised divergence dates also have bearing on the status of the African forest elephant. Based on nuDNA sequences, Roca et al. [6] argued that the African forest elephant represents its own species, *L. cyclotis,* distinct from the savannah elephant, L. africana. Using nuDNA sequence data and a divergence time between African and Asian elephants of 5 mya, they estimated the divergence between the nuDNA sequences from African savannah and forest elephants to be 2.63 ± 0.94 mya, and argued that both the deep divergence and the reciprocal monophyly between forest elephants and savannah elephants with regard to nuDNA support the distinction of the two forms as different species, a view also supported by microsatellite analyses [39]. However, based on extensive mtDNA analyses, both Eggert et al. [40] and Debruyne [7] disputed this view, as they found forest and savannah elephants being polyphyletic with respect to mtDNA sequences. In an extension of their earlier analysis, using both X and Y chromosomal and mtDNA sequences, Roca et al. [41] confirmed their view of a distinction on the species level and argued for unidirectional gene flow of mtDNA from forest elephants to savannah elephants. Although, complete mtDNA sequences for the two African elephant are not available, we can use our results to recalculate the divergence time between forest and savannah elephant inferred from the nuclear sequences. Using our estimate of 7.6 mya for the initial *Loxodonta* divergence increases the estimated divergence time to 4.0 mya. This date is older than the divergence times of many species pairs and hence supports the classification of African savannah and forest elephants as different species as proposed by Roca et al. [6,41].

### mtDNA Substitution Rate

The initial sequencing of the mammoth mitochondrial genome indicated that the substitution rate within Elephantidae is much lower than in humans and the African great apes [3]. We can use the mastodon sequence to determine whether this arose recently or in the more distant past. We compared the substitution rates of the mammoth, elephants, and mastodon, which had a most recent common ancestor 24–28 mya, to those of human, chimpanzee, gorilla, and baboon, which had a most recent common ancestor about 33 mya [42]. The substitution rate for the whole mtDNA genome was found to be more than twice as high in the four species of primates than in the four species of proboscideans. However, the distribution of rates among sites within the genome was almost identical (Table S3). It is not clear what causes the difference in rates. A possible explanation could be differences in body size, which in turn influence metabolic rates [43,44], but further studies on more species would be necessary to evaluate whether this is the major, or only, cause.

### Conclusions

The sequence of a complete mtDNA genome obtained from a mastodon tooth extends the time frame for large-scale sequencing of ancient DNA substantially. Inclusion of this sequence in phylogenetic analyses confirms mammoth and Asian elephants as sister taxa and provides evidence for earlier divergences between Elephantidae species. The similarity of the divergence dates between Elephantidae species and between humans and African great apes suggests that a change in environmental conditions triggered speciation in African mammals beginning some 7.5–8 mya. Finally, we found further evidence that the mitochondrial substitution rate in proboscideans is considerably lower than in primates, and this difference manifested by at least 24 mya.

Although permafrost environment is especially suitable for long-term DNA survival, DNA sequences about 130,000 y old have also been reported from non-permafrost remains. Moreover, the age limit for preservation of plant DNA in permafrost environment is even older, currently at 300,000–500,000 y [45]. Even if the DNA from specimens is more fragmented than in the sample used in this study, the two-step multiplex procedure would allow reconstruction of long, continuous sequences such as complete mitochondrial genomes, substantially enlarging the possibilities of ancient DNA analyses.

## Materials and Methods

### Specimen provenance.

For DNA extraction, we cut a ∼200-g sample along the tooth root of a mastodon molar (United States Bureau of Land Management–Alaska collection IK-99–237) collected in 1999 on the Ikpikpuk River (69° 22′ 10′′ N, 154° 40′ 46′′ W; Figure 1), which drains the central arctic coastal plain of northern Alaska. The tooth was a detrital find collected on a point bar along with numerous other large mammal bones of late Pleistocene affinity. These bones are eroded from cutbanks and deposited in large quantities on point bars along a number of rivers that drain the arctic coastal plain. The fluvial and alluvial sediments in these systems were mostly deposited during the late Pleistocene and early Holocene, but contain reworked bones of Pleistocene age [46–48]. There is a rare occurrence of sediments dating to the last interglacial period (∼130,000–100,000 y BP). To date, an assemblage of ∼3,000 bones has been collected, 312 of which have been radiocarbon-dated [48,49]. Most are within the radiocarbon range (<50,000–40,000 y), but about 20% have returned nonfinite dates (i.e., “greater than” ages ranging from 50,000 to 40,000 y BP). Radiocarbon analyses of a collagen extract from mastodon tooth IK-99–237 yielded a nonfinite ^14^C age of >50,000 y BP (Lawrence Livermore National Laboratory number CAMS91805), which places a lower limit on its age. In terms of its maximum age, there are only two bones in the entire assemblage from a taxon *(Praeovibos)* that dates to the middle Pleistocene, and even those two bones were re-worked into late Pleistocene sediments. Consequently, it is conservatively estimated that nonfinite-age bones in the assemblage date to between approximately 50,000 y BP and the end of the penultimate glaciation (about 150,000 y ago). Since the mastodon is principally an inhabitant of interglacial periods in Alaska, it is most likely IK-99–237 dates to the last interglacial period (i.e., its maximum age is probably ∼130,000 y BP), though we have no way of absolutely ruling out an age between 100,000 and 50,000 y BP. Bones in this assemblage are extremely well-preserved because they were entombed in permafrost sediments before being exposed by river erosion. This accounts for the exceptional preservation of DNA in IK-99–237.

### DNA extraction.

Part of the tooth root was cut into small pieces and ground to a fine powder in a freezer mill (freezer mill 6750, Spex SamplePrep, http://www.spexcsp.com/sampleprep/) using liquid nitrogen. About 25 g of the powder was incubated overnight under constant agitation at room temperature in 700 ml of extraction buffer consisting of 0.45 M EDTA (pH 8.0) and 0.25 mg/ml proteinase K . After centrifugation the supernatant was concentrated to ∼50 ml using the Vivaflow 200 system (Vivascience, http://www.vivascience.com) with a polyethersulfone membrane with a molecular weight cut-off of 30,000 [50]. DNA was bound to silica using 40 ml of guanidinium thiocyanate buffer [51] and 100 μl of silica suspension for each of the five 10-ml aliquots of concentrated extraction buffer, with the pH adjusted to 4.0 using hydrochloric acid. After incubation for 3 h under constant agitation the silica was pelleted by short centrifugation and washed once with 1 ml of guanidinium thiocyanate buffer and twice with 1 ml of wash solution (51.3% ethanol, 125 mM NaCl, 10 mM Tris, and 1 mM EDTA [pH 8.0]). DNA was eluted using 50 μl of TE buffer for each of the five aliquots, resulting in ∼250 μl of extract. An extraction blank was carried alongside throughout all steps of extraction to monitor for possible contamination.

### Multiplex amplification and sequencing.

We designed 78 primer pairs using published sequences of African (Loxodonta1) and Asian (Elephas1) elephants, mammoth (Mammuthus1), and dugong (Dugong). The length of the targeted fragments varied between 139 and 334 bp (including primer), covering the entire mitochondrial genome of the mastodon except a repeat sequence in the control region.

Concordant with the initial paper describing the multiplex approach for DNA amplification from ancient samples [3], we divided the primer pairs for the first step into two sets to avoid amplification of the short overlapping fragments between adjacent amplification products [52]. In the second step, each primer pair was used individually, and to increase the specificity of amplification, in all except four cases, one primer per pair was “nested” compared to the first step, resulting in fragments between 139 and 324 bp long (including primers). Wherever possible, primers were designed to exclude amplification of human DNA.

Four primary amplifications (the multiplex step) were done for each of the two primer sets using two times 5 μl of 1:5 and 1:10 dilutions, respectively, of the extract, resulting in altogether eight primary amplifications. PCRs were conducted in a final volume of 20 μl consisting of 1× GeneAmp PCR Buffer II (Applied Biosystems, http://www.appliedbiosystems.com), 4 mM MgCl_2_, 1 mg/ml BSA, 250 nM of each dNTP, 2 U AmpliTaq Gold (Applied Biosystems), and 150 nM each of 78 primers (39 primer pairs per set). Initial denaturation and activation of the polymerase was done for 9 min at 94 °C followed by 30 cycles at 94 °C for 20 s, at 48, 50, or 52 °C for 30 s, and at 72 °C for 30 s. Aliquots of 5 μl of a 1:20 dilution of this primary amplification were used for the two sets of 39 individual secondary amplifications. These secondary PCRs were done in a final volume of 20 μl consisting of the same reagent concentrations as described above except that only 0.25 U of AmpliTaq Gold was used and the primer concentration was raised to 1.5 μM. Cycling conditions were the same as above except that between 30 and 40 cycles were performed. Extraction and water controls were carried along during all steps.

As not all amplifications were successful in the first attempt, several primers were redesigned after sequencing the flanking regions obtained from successful amplifications, and more primary amplifications were performed (see Results/Discussion and Protocol S1).

Amplification products were visualized on 2.0% agarose gels, and products of correct lengths were cloned using the Topo TA Cloning Kit (Invitrogen, http://www.invitrogen.com). In cases where amplifications showed visible primer dimers in addition to products of the correct length, the products were isolated from the gel and purified using the QIAquick Gel Extraction Kit (Qiagen, http://www.qiagen.com). After colony PCR [53] and purification of the products using the QIAquick PCR Purification Kit (Qiagen) and a Biorobot, a minimum of three clones per amplification (see Protocol S1) were sequenced on an ABI 3730 capillary sequencer (Applied Biosystems) using M13 universal primers.

### Phylogenetic analyses.

All alignments were made using ClustalW [54] with default parameters. The D-loop sequences were removed from all genome sequences prior to analyses. We aligned the mastodon sequence to previously published sequences of two mammoths (Mammuthus1 and Mammuthus2), two African elephants (Loxodonta1 and Loxodonta2), and two Asian elephants (Elephas1 and Elephas2). For this alignment, we verified that the start and stop codons were aligned and that the open reading frames were preserved. For the concatenation of the protein coding genes, all genes were aligned individually and subsequently concatenated, ignoring the fact that some of them were overlapping. Hence, some nucleotides are duplicated in our concatenation. We examined publicly available mastodon sequences by aligning them with our sequence and constructing NJ trees using MEGA3.1 [55] with default parameters (see Protocol S2 for details on the program settings).

To test the suitability of our outgroup we estimated the degree of substitution saturation for the concatenated protein coding genes by computing the number of synonymous and nonsynonymous substitutions per site with the Pamilo-Bianchi-Li method implemented in MEGA3.1. We also used MEGA3.1 to compute the number of substitutions per site between proboscideans, dugong, and hyrax and to compute the transition/transversion ratio by aligning the seven proboscidean mtDNA genomes with those of dugong, hyrax, cow, and ostrich.

To solve the Elephantidae phylogeny we used MEGA3.1 to reconstruct trees using NJ and maximum parsimony. Initially, parameters similar to those used in [4] were chosen. We used the default parameters from MEGA3.1 with 10,000 bootstrap replicates. In addition, we built a Bayesian tree using MrBayes 3.1 [56]. The tree with maximum posterior probability was computed using one million iterations (see Protocol S1 for information on the options used). A GTR model [57,58] and a HKY85 model [59] of nucleotide substitution, both with gamma distributed rates of substitutions among sites, were used. The ML tree was constructed using Paup* [60] with an exhaustive search. For the ML tree, we performed 1,000 bootstrap replicates and chose a GTR model of substitution with gamma distributed rates among sites. Finally, we reconstructed the phylogeny for the seven sequences individually for each protein coding gene using MrBayes 3.1 and a HKY85 model of substitutions as well as by NJ as described above. The same analysis was performed for the concatenated sequence of the protein coding genes. However, the result of this analysis did not differ significantly from that of the analysis performed using the complete sequence (results not shown).

Because the different reconstructions revealed a single topology, we tested the different models of nucleotide substitutions against each other following the Felsenstein hierarchy in order to choose the best-fitting model. To this end, we compared the different models by likelihood ratio tests with the program baseml in PAML [61] using the alignment of the seven sequences without the D-loop, using gamma distributed rates among sites with eight categories, and considering a non-clock situation. The topology of the tree used for these analyses is shown in Figure 5 and identical to that in previous publications [3,4]. We reconstructed the tree once again using the above methods with TN93, the simplest model of substitution for which none of the more complex models fitted the data significantly better, but no significant difference from the previous results was observed (results not shown). To test whether the seven sequences evolved following a molecular clock we estimated the ML values for the phylogenetic tree of the seven sequences under both a non-clock assumption and a molecular clock assumption and compared the likelihoods of the trees obtained under the two assumptions using a likelihood ratio test (five degrees of freedom). As before, we assumed gamma distributed rates among sites and TN93 as a model of substitution.

Given the difficulty of precisely estimating calibration points and hence divergence times [62,63], we used a Bayesian approach and the program mcmctree to estimate the posterior means and the CIs of the divergence times. We used as a calibration point the divergence of the mastodon, estimated to be 24–28 mya [31] applying the lower and upper bound method. Given the unavailability of TN93, we chose HKY85, the most complex model implemented in mcmctree. The burn-in was set to 10,000, the number of samples to 100,000, and the sample frequency to five, as in [30], with four independent chains for each analysis. We chose wide priors for κ, the transition/transversion ratio, *r,* the substitution rate, and α, the parameter of the gamma distribution of rate variation among sites, to avoid too strong an influence of the priors on the posteriors. The 95% prior intervals were set to (0.00, 111.24), (0.02, 3.69), and (0.00, 1.23) for κ, *r,* and α, respectively. To compute the rate of substitution for primates we used the same approach for a four-taxon tree of baboon, gorilla, chimpanzee, and human. The calibration point was set to a lower and upper bound of 6–8 mya for the chimpanzee–human split [64] and an upper bound of 33 mya for the baboon divergence ([42] and references therein). We used the same disperse priors as before for κ, *r,* and α and the same options for the Markov chain Monte Carlo.

Since the use of a single model of evolution for the whole mtDNA sequence may result in errors, we partitioned the data into five subsets. We separated the first, second, and third positions of the codons, the *rRNAs,* and the *tRNAs*. The few noncoding sites were excluded to avoid overparametrization. The protein–protein overlapping fragments were classified under the second codon position, except for the seven sites between *ND5* and *ND6* that were excluded because the two genes are on different strands. Indeed we inverted the strands according to the transcription process. The phylogenetic tree was constructed using MrBayes 3.1 with a GTR model of substitution and one million iterations with unlinked partitions. We also allowed the partitions to have different rates. The divergence times using the partitions were computed using mcmctree with the options described above.

## Supporting Information

Figure S1 Trees Including Previously Published Mastodon Sequences (A) NJ tree (MEGA3.1 default parameters; 10,000 replicates for the bootstrap) for the fragments of 228 bp. The sequences starting with “CYTB” are the usual sequences used in this study.
(B) NJ tree (MEGA3.1 default parameters; 10,000 replicates for the bootstrap) for the fragment of 294 bp. The sequences starting with “CYTB” are the usual sequences used in this study.
(924 KB TIF)

Figure S2 Click here for additional data file. Sampling Properties of the Whole Genome on the Inferred Phylogeny
(A) Results for sliding window analyses. The different trees are color coded, and the bootstrap support (10,000 replicates) is indicated on the *y*-axes (for the unresolved topology we assigned a value of 50). Position 1 on the *x*-axis corresponds to the first base pair of tRNA-Phe. As can be seen from the results, up to a window size of 3,000 bp, topologies that differ from the whole genome tree are obtained, and even with 5,000 bp, the topology of the tree is often unresolved. We used only one sequence per species for this analysis (i.e., Elephas1, Loxodonta1, Mammuthus1, and the mastodon sequence). The total length of the alignment is 15,431 bp because the D-loop region has been removed.
(B) Proportion of phylogenetic trees that yield the whole genome tree topology (*y*-axis) for contiguous sequences and for random sampling.
(390 KB TIF)

Figure S3 Saturation of the Number of Substitutions When Using the Hyrax and Dugong as Outgroups (3.4 MB TIF)

Protocol S1 Sequence Determination (35 KB DOC)

Protocol S2 Sampling Properties of the Whole Genome on the Inferred Phylogeny (25 KB DOC)

Protocol S3 Supporting Methods for the Phylogenetic Analyses: Options for the Programs Used: MrBayes 3.1, Paup*, and PAML (43 KB DOC)

Table S1 Pairwise Number of Substitutions for Whole Mitochondrial Genomes without D-Loop for Asian Elephant, African Elephant, Mammoth, Mastodon, Dugong, and Hyrax (32 KB DOC)

Table S2 Gene Trees: Phylogeny Gene by Gene of the Elephantids Using Bayesian Inference (HKY85) or NJ (K80) (39 KB DOC)

Table S3 Difference in Rates of Substitution between Four Species of Primates and Four Species of Proboscideans (33 KB DOC)

Table S4 Posterior Probabilities and CIs of the Divergence Times for the Partitioned Data (35 KB DOC)

### Accession Numbers

The GenBank (http://www.ncbi.nlm.nih.gov) accession numbers for the complete mitochondrial genome sequences of *E. maximus, L. africana,* and Mammuthus primigenius are NC_005129.1 for Elephas1 and NC_005129.2 for Elephas2, NC_000934.1 for Loxodonta1 and DQ316069.1 for Loxodonta2, and NC_007596.2 for Mammuthus1 and DQ316067.1 for Mammuthus2. For the partial sequences of Mammut americanum the GenBank accession numbers are U23737.1 (denoted Yang Mastodon), AY028924.1, and AF279699.1; for the partial sequences of Mammuthus primigenius the GenBank accession numbers are U23738.1 (denoted Yang Mammuthus1) and U23739.1 (denoted Yang Mammuthus2). The accession number for the whole mitochondrial genome of Mammut americanum determined in this study is EF632344. For the non-proboscideans, the GenBank accession numbers for the whole genomes used in this study are NC_003314.1 for D. dugon (Dugong), NC_004919.1 for P. capensis (Procavia), NC_006853.1 for B. taurus (Bos), Y12025.1 for S. camelus (Struthio), NC_001992.1 for Papio hamadryas (Baboon), NC_001645.1 for Gorilla gorilla (Gorilla), NC_001643.1 for Pan troglodytes (Chimpanzee), and NC_001807.4 for Homo sapiens (Human)*.*

## Abbreviations

BP: before present
CI: credibility interval
ML: maximum likelihood
mtDNA: mitochondrial DNA
mya: million years ago
NJ: neighbor joining
nuDNA: nuclear DNA
