# Supplementary material for: Proboscidean Mitogenomics: Chronology and Mode of Elephant Evolution Using Mastodon as Outgroup
Source: PLoS Biol. 2007 Jul 24;5(8):e207. doi: 10.1371/journal.pbio.0050207 (PMC1925134; doi:10.1371/journal.pbio.0050207)
Supplement: Supplementary file 1 [file pbio.0050207.sd001.doc]

**Protocol S1.** Sequence Determination

To determine the mastodon mtDNA genome sequence, we cloned altogether 218 PCR products. To obtain a consensus sequence for each fragment, we sequenced at least 3 clones per amplification product. However, for 128 PCR products we sequenced 4 clones, for 64 products more than four clones and only for 26 products the minimum number of 3 clones. We would like to note, though that 3 clones allow the reconstruction of a consensus sequence for a certain PCR product. More importantly, each nucleotide position was determined from at least two independent primary PCRs and a substantial fraction of the positions was determined from three or more primary amplifications.

In the 218 amplification products sequenced, we observed 66 consistent changes, i.e. positions at which all clones from one amplification are identical but differ from all clones from a second amplification [1,2]. To determine the correct sequence for such positions we amplified, cloned and sequenced affected fragments a third time and generally accepted the nucleotide observed in two of the three amplifications. As cytosine deamination, resulting in C to T or G to A substitutions in PCR amplified ancient DNA sequences is known to be the by far most prevalent type of DNA damage [1,3,4,5], we performed additional primary PCRs in cases when two of three PCRs showed a T or A, respectively, with the minority nucleotide being a C or G. Consistent changes were observed in 27 primary amplifications obtained from 19 different fragments. Interestingly, at four positions, a T or A rather than a C or G were found to be the majority nucleotide after three independent PCRs, contrary to the expectation based on our knowledge about ancient DNA damage. In each case we sequenced clones from at least two add primary PCRs showing cytosine to be the correct nucleotide in all four cases. Thus, all 66 consistent changes were found to be C to T or G to A substitutions, consistent with cytosine deamination as the prevalent type of miscoding lesion in ancient DNA. Given that the fragments showing consistent changes contain 1584 cytosine and guanine positions and assuming that the 66 consistent changes are randomly distributed, observing four positions to be hit twice is not unlikely (chi-square = 5.8; p = 0.056).

References

1. Hofreiter M, Jaenicke V, Serre D, Haeseler Av A, Pääbo S (2001) DNA sequences from multiple amplifications reveal artifacts induced by cytosine deamination in ancient DNA. Nucleic Acids Res 29: 4793-4799.

2. Handt O, Krings M, Ward RH, Pääbo S (1996) The retrieval of ancient human DNA sequences. Am J Hum Genet 59: 368-376.

3. Gilbert MT, Hansen AJ, Willerslev E, Rudbeck L, Barnes I, et al. (2003) Characterization of genetic miscoding lesions caused by postmortem damage. Am J Hum Genet 72: 48-61.

4. Hansen A, Willerslev E, Wiuf C, Mourier T, Arctander P (2001) Statistical evidence for miscoding lesions in ancient DNA templates. Mol Biol Evol 18: 262-265.

5. Poinar HN, Schwarz C, Qi J, Shapiro B, Macphee RD, et al. (2006) Metagenomics to paleogenomics: large-scale sequencing of mammoth DNA. Science 311: 392-394
