# Supplementary material for: Proboscidean Mitogenomics: Chronology and Mode of Elephant Evolution Using Mastodon as Outgroup
Source: PLoS Biol. 2007 Jul 24;5(8):e207. doi: 10.1371/journal.pbio.0050207 (PMC1925134; doi:10.1371/journal.pbio.0050207)
Supplement: Supplementary file 2 [file pbio.0050207.sd002.doc]

**Protocol S2.** Sampling Properties of the Whole Genome on the Inferred Phylogeny

We tested which length of sequence is necessary to obtain the same phylogenetic tree as with the complete mtDNA genome sequence. To that end we used a sliding window analysis with window sizes of 500, 1000, 3000, 5000, 8000, and 10000 bp, respectively, and steps of 10 bp, and constructed neighbor joining trees using Paup* with default parameters. The results show, that from 8000 bp onwards, only the whole genome tree is obtained, whereas below this window size, different topologies are obtained as well, and up to a window size of 3000 bp with relatively high bootstrap values (Fig. S2a).

We also used a random sampling procedure without replacement to obtain phylogenetic trees from non-contiguous sequences, as Cummings et al. [1] found that contiguous sequences are more likely to yield incorrect trees. However, when we compared the sliding window analysis to a random sampling, we found that they both yield similar proportion of trees identical to the whole genome tree (Figure S2b). We cannot reject independence of sites as Cummings et al On the other hand sequence length in itself is crucial for obtaining whole genome topologies.

Reference

1. Cummings MP, Otto SP, Wakeley J (1995) Sampling properties of DNA sequence data in phologenetic analysis. Molecular Biology and Evolution 12: 814-822.
