# Supplementary material for: Proboscidean Mitogenomics: Chronology and Mode of Elephant Evolution Using Mastodon as Outgroup
Source: PLoS Biol. 2007 Jul 24;5(8):e207. doi: 10.1371/journal.pbio.0050207 (PMC1925134; doi:10.1371/journal.pbio.0050207)
Supplement: Supplementary file 3 [file pbio.0050207.sd003.doc]

**Protocol S3**. Supporting methods for the phylogenetic analyses: options for the programs used.

**MrBayes3.1**

All trees constructed with MrBayes3.1 [1] were obtained with 1 million generations for the chains, a sample frequency of a 100 and a burnin of 2*’500* (ngen=1000000; Samplefreq=100; burnin=2*’*500). The site by site rate variation was set to a gamma distribution (rates=gamma) for all the Bayesian trees and an HKY85 [2] (nst=2) or GTR [3,4] (nst=6) model of substitution were chosen.

The tree for the combined partition of the whole genome was constructed assuming that the partitions are unlinked (unlink shape=(all) statefreq=(all) revmat=(all)) and the they have independent rates (prset applyto=(all) ratepr=(all)).

**Paup***

The maximum likelihood tree for the whole genome was constructed using Paup* [5] with an exhaustive search (allsearch) assuming a GTR [3,4] (nst=6; rmatrix=estimate) or a TN93 (nst=6 rmatrix=estimate rclass=(a b a a c a)) [6] model of substitutions, with a gamma distribution of rates among sites (rates=gamma ncat=8), no molecular clock (clock=no) and 1*’*000 replicates for the bootstrap (bootstrap nreps=1000).

**PAML**

The program **baseml** of the package PAML [7] was used to choose the best fitting model. We assumed no molecular clock (clock = 0), a gamma distributed rates among sites (fix_alpha = 0; ncatG = 8), no fixed κ (fix_kappa = 0). The three last parameters were kept fixed for all the subsequent ML analyses. Multiple runs (to avoid local maxima) with different starting values were performed for each of the model of substitution implemented in baseml (model = 0 1 2 3 4 5 6 7 8) with base frequencies estimated by maximum likelihood (nhomo = 1) whenever possible. This gave rise to 12 likelihood ratio tests according to the nesting relationship between models.

A molecular clock was tested for the whole genome by using the same parameters as before with a TN93 model of substitution (model = 6; nhomo = 1) by comparing the likelihoods when a clock was assumed (clock = 1) or when no clock (clock = 0) was assumed.

The divergence times using the 6 my Asian-African elephant split were computed with baseml by assuming a clock (clock = 1) using TN93 as a model of substitution (model = 6; nhomo = 1) and by specifying the calibration point in the rooted tree (‘@0.06’).

The divergence times when using the 24-28 my mastodon divergence as a calibration point were computed with **mcmctree** with the lower and upper bound method (‘>0.24 <0.28’). The MCMC parameters were set to 100’000 generations for the chains, 5 for the frequency of sample and 10’000 for the burnin (nsample = 100000; sampfreq = 5; burnin = 10000) and all analyses were made with HKY85 [2] as a model of substitution. The birth death and sampling parameters were set to 2, 2 and 0.01 (BDparas = 2 2 0.1); different sets of parameters were tried but this did not affect the posterior estimates significantly. The fine-tuning parameters were chosen to be 0.2, 0.4, 0.5 and 0.2 for the times, substitution rates, mixing, and substitution parameters (finetune = 0.2 0.4 0.5 0.2) for the whole genome analysis to keep the acceptance rate between 10 and 80%. For the same reasons for the partition (ndata = 5) and the primates dataset, these parameters were set to finetune = 0.2 0.4 0.5 0.3 and finetune = 0.2 0.5 0.5 0.1, respectively. The shape and inverse scale parameters for the priors for κ, α and r were set to (0.4, 0.02), (1, 3) and (1, 1) (kappa_gamma = 0.4 0.0.2; alpha_gamma = 1 3; rgen_gamma = 1 1) for all runs. A particularly disperse prior was chosen for κ because the posterior distributions were particularly sensitive to the choice of this prior. For the primates dataset we used two calibration points: a lower and upper bound for the chimpanzee human divergence of 6-8 my (‘>0.06 <0.08’) and an upper bound for the baboon divergence of 33 my (‘<0.33’) [8] and references therein). Finally each analysis was conducted by running the chain at least 4 times, using different starting values (negative seed) to confirm consistency between runs.

References

1. Ronquist F, Huelsenbeck JP (2003) MrBayes 3: Bayesian phylogenetic inference under mixed models. Bioinformatics 19: 1572-1574

2. Hasegawa M, Kishino H, Yano T (1985) Dating of the human-ape splitting by a molecular clock of mitochondrial DNA. J Mol Evol 22: 160-174

3. Lanave C, Preparata G, Saccone C, Serio G (1984) A new method for calculating evolutionary substitution rates. J Mol Evol 20: 86-93

4. Tavare S (1986) Some probabilistic and statistical problems in the analysis of DNA sequences. Lectures on the Mathematics in the Life Sciences 17: 57-86.

5. Swofford DL (2002) Paup*. Phylogenetic Analysis Using Parsimony (*and Other Methods). Version 4.

6. Tamura K, Nei M (1993) Estimation of the number of nucleotide substitutions in the control region of mitochondrial DNA in humans and chimpanzees. Mol Biol Evol 10: 512-526

7. Yang Z (1997) PAML: a program package for phylogenetic analysis by maximum likelihood. Comput Appl Biosci 13: 555-556

8. Steiper ME, Young NM, Sukarna TY (2004) Genomic data support the hominoid slowdown and an Early Oligocene estimate for the hominoid-cercopithecoid divergence. Proc Natl Acad Sci U S A 101: 17021-17026.
