# Supplementary material for: Proboscidean Mitogenomics: Chronology and Mode of Elephant Evolution Using Mastodon as Outgroup
Source: PLoS Biol. 2007 Jul 24;5(8):e207. doi: 10.1371/journal.pbio.0050207 (PMC1925134; doi:10.1371/journal.pbio.0050207)
Supplement: Supplementary file 4 [file pbio.0050207.st001.doc]

**Table S1.** Pairwise number of substitutions for whole mitochondrial genomes without Dloop of the Asian elephant, the African elephant, the mammoth, the mastodon, the dugong and the hyrax.

**Elephas 1 Elephas2 Loxo 1	Loxo 2	Mamm 1 Mamm 2 Masto	Dugo	Pro**
