# Supplementary material for: Proboscidean Mitogenomics: Chronology and Mode of Elephant Evolution Using Mastodon as Outgroup
Source: PLoS Biol. 2007 Jul 24;5(8):e207. doi: 10.1371/journal.pbio.0050207 (PMC1925134; doi:10.1371/journal.pbio.0050207)
Supplement: Supplementary file 5 [file pbio.0050207.st002.doc]

**Table S2.** Gene trees. Phylogeny gene by gene of the elephantids using Bayesian inference (HKY85) or neighbor joining (K80). The posterior probabilities and bootstrap values are shown in parenthesis.
