# Supplementary material for: Proboscidean Mitogenomics: Chronology and Mode of Elephant Evolution Using Mastodon as Outgroup
Source: PLoS Biol. 2007 Jul 24;5(8):e207. doi: 10.1371/journal.pbio.0050207 (PMC1925134; doi:10.1371/journal.pbio.0050207)
Supplement: Supplementary file 6 [file pbio.0050207.st003.doc]

**Table S3.** Difference in rates of substitution between four species of primates and four species of proboscideans.

|  | Proboscideans | | Primates | |
| --- | --- | --- | --- | --- |
|  | Post mean | 95% CI | Post mean | 95% CI |
| r(10^-8^ subst/nt/year) | 0.42 | (0.36, 0.49) | 1.14 | (1.02, 1.26) |
| κ | 39.8 | (33.8, 46.8) | 20.3 | (17.8, 23.0) |
| α | 0.22 | (0.19, 0.26) | 0.22 | (0.20, 0.24) |

For the proboscideans, the calibration point used is the mastodon divergence estimated to be 24-28 my [1,2]. For the primates two calibration points were used: the human chimp divergence set to be between 6 and 8 my [3] and the baboon divergence set to younger than 33 my ([4] and references therein).

References

1. Shoshani J, Golenberg EM, Yang H (1998) Elephantidae phylogeny: morphological versus molecular results. Acta Theriologica Suppl 5: 89-122.

2. Shoshani J, Walter RC, Abraha M, Berhe S, Tassy P, et al. (2006) A proboscidean from the late Oligocene of Eritrea, a "missing link" between early Elephantiformes and Elephantimorpha, and biogeographic implications. Proceedings of the National Academy of Sciences of the United States of America 103: 17296-17301.

3. Brunet M, Guy F, Pilbeam D, Mackaye HT, Likius A, et al. (2002) A new hominid from the Upper Miocene of Chad, Central Africa. Nature 418: 145-151.

4. Steiper ME, Young NM, Sukarna TY (2004) Genomic data support the hominoid slowdown and an Early Oligocene estimate for the hominoid-cercopithecoid divergence. Proc Natl Acad Sci U S A 101: 17021-17026.
