# Supplementary material for: Proboscidean Mitogenomics: Chronology and Mode of Elephant Evolution Using Mastodon as Outgroup
Source: PLoS Biol. 2007 Jul 24;5(8):e207. doi: 10.1371/journal.pbio.0050207 (PMC1925134; doi:10.1371/journal.pbio.0050207)
Supplement: Supplementary file 7 [file pbio.0050207.st004.doc]

**Table S4.** Posterior probabilities and CI of the divergence times for the partitioned data. The same approach was used as for the whole genome. Each partition can evolve under different rates, transition transversion ratio and the variation of rates among sites can be different too. As in Table 2, the node t_1_ corresponds to the mastodon divergence and is our calibration point (set to 24-28 my). The node t_2_ is the African elephant-Asian elephant divergence, t_3_ is the mammoth divergence. The nodes t_4_, t_5_, t_6_ are the nodes for the intra species divergence for the Asian elephant, the mammoth and the African elephant respectively.

|  | Post mean (95% CI) | | | | |
| --- | --- | --- | --- | --- | --- |
|  | rRNA (2532bp) | tRNA (1499bp) | 1st (3762bp) | 2^nd^ (3807bp) | 3rd (3754bp) |
| t_1_ (my) calibration | 25.8 (24.0, 28.0) | | | | |
| t_2_(my) | 7.5 (6.5, 8.5) | | | | |
| t_3_(my) | 6.5 (5.7, 7.5) | | | | |
| t_4_(my) | 1.3 (1.1, 1.6) | | | | |
| t_5_(my) | 0.27 (0.18, 0.37) | | | | |
| t_6_(my) | 0.14 (0.08, 0.21) | | | | |
| r(10^-8^ subst/nt/year) | 0.15 (0.12, 0.19) | 0.22 (0.16, 0.30) | 0.28 (0.24, 0.34) | 0.09 (0.07, 0.11) | 1.22 (1.04, 1.42) |
| κ | 24.3 (14.8, 39.01) | 56.8 (28.3, 40.47) | 29.3 (21.0, 40.5) | 20.1 (11.8, 33.15) | 61.3 (49.8, 74.9) |
| α | 0.10 (0.02, 0.21) | 0.14 (0.07, 0.27) | 0.14 (0.10, 0.20) | 0.07 (0.00, 0.20) | 2.08 (1.45, 3.03) |
